# Dress Syndrome in 7-year-old Male Child – Case Report

**DOI:** 10.34763/jmotherandchild.20202403.2019.d-20-00006

**Published:** 2021-01-12

**Authors:** Monika Prylińska, Marta Dworakowska-Kicińska, Aneta Krogulska

**Affiliations:** 1 Department of Pediatrics, Allergology and Gastroenterology Collegium Medicum in Bydgoszcz, Nicolaus Copernicus University in Toruń, Bydgoszcz, Poland

**Keywords:** child, DRESS, drug reaction with eosinophilia and systemic symptoms, drug-induced hypersensitivity, paediatric

## Abstract

Drug rash with eosinophilia and systemic symptoms (DRESS) is a severe drug-induced hypersensitivity reaction, which, due to the asymptomatic beginning and non-specific nature of symptoms, is hard to identify. This report presents the case of a 7-year-old boy, who was referred to the Department of Paediatric Surgery with fever up to 38°C, vomiting and diarrhoea, accompanied by erythematous, maculopapular rash. Based on laboratory and radiology tests and specific diagnostic criteria, DRESS syndrome was diagnosed. The presented case report emphasises the need to carry out differential diagnosis, including the potentially life-threatening DRESS syndrome, with common symptoms in children such as fever and rash.

## Introduction

Drug rash with eosinophilia and systemic symptoms (DRESS) syndrome encompasses drug rash with eosinophilia and organ impairment symptoms. It was first described in 1996 by Bocquet et al. (1). The name was coined by Callot et al. (2) in a study of 21 patients with acute organ disease, eosinophilia and skin symptoms. The frequency of DRESS is estimated to be between 1/1,000 and 1/10,000 patients, and although this frequency may well be higher, it is rarely diagnosed (3). The pharmacological compounds commonly associated with DRESS syndrome are phenytoin and phenobarbital (4).

The clinical symptoms of DRESS syndrome appear 2–8 weeks after taking the reaction-inducing drug; they typically manifest as fever, maculopapular rash, peripheral lymphadenopathy, haematological disorders and impairment of one or more organs (3). Mortality is estimated to be 10% in adults (4) and 5.4% in children (5).

## Case report

A 7-year-old boy, M.C., was referred to the Department of Pediatrics, Allergology and Gastroenterology in Bydgoszcz with a rash and fever. On interviewing, we found that he had attended the Department of Paediatric Surgery 3 weeks previously for the treatment of appendicitis complicated by peritonitis. One week after appendectomy, he required re-laparotomy for bowel obstruction. Intensive parenteral antibiotic therapy was applied: cefotaxime, metronidazole and amikacin initially, followed by vancomycin, imipenem with cilastatin and fluconazole. After clinical improvement and normalisation of the inflammatory markers, he was discharged in good condition.

The next day, the boy experienced vomiting and diarrhoea (up to six loose stools per day). Three days later, he experienced a 38°C fever accompanied by erythematous rash covering the earlobes, face and torso. Therefore, the next day, the boy was readmitted to hospital for further diagnosis and treatment.

Upon admission, the patient was assessed to be in good general condition. Apart from the surgical scar in the abdominal area, provided with stitches, physical examination revealed an intense red, maculopapular rash throughout the body, being most severe on the face. Laboratory tests revealed eosinophilia (21%) and elevated C-reactive protein (CRP; 67 mg/L). Initially, rupatadine was administered. In the following days, fever up to 39°C, diarrhoea and rash with a tendency to merge were observed. Additionally, the boy developed swelling of the subcutaneous tissue, with the most intense skin lesions being observed on the cheeks, earlobes and lips. The following day, an increase in both inflammatory parameters and eosinophilia was observed. The results of all the laboratory tests are presented in Table 1.

**Table 1:** The results of laboratory tests during hospitalisation

		Results of the tests				
Laboratory test	Laboratory norm	Day of hospitalisation				
		1	3	5	7	16
CRP (mg/L)	<5.0	67.5	126.45	47.44		0.83
Procalcitonin (ng/mL)	<0.5	0.81	8.09			0.4
Eosinophilia (%)	2–4	21	30	36		0.56
Haemoglobin (g/dL)	12.0–15.0	11.1	10.1		8.6	9.7
APTT (sec)	27–40		47.2		53.5	32.5
D-Dimers (ng/mL)	<500		1510	2578		900
Albumin (g/dL)	3.8–5.4		4.3		3.0	4.5
Protein in the urine (mg/dL)	Not present		10	50		Not present
Creatinine (mg/dL)	0.3–0.7			1.13	1.08	0.54
ALT (U/L)	<44			12	12	12
AST (U/L)	<48			18	12	14
TSH (mIU/mL)	0.3500– 4.9400		5.9817			
fT4 (ng/dL)	0.70–1.48		1.26			
fT3 (pg/mL)	1.71–3.71		2.18			

CRP, C-reactive protein; APTT, activated partial thromboplastin time; ALT, alanine aminotransferase; AST, aspartate aminotransferase; TSH, thyroid-stimulating hormone; fT3, free tri-iodothyronine; fT4, free thyroxine.

**Figure 1:**
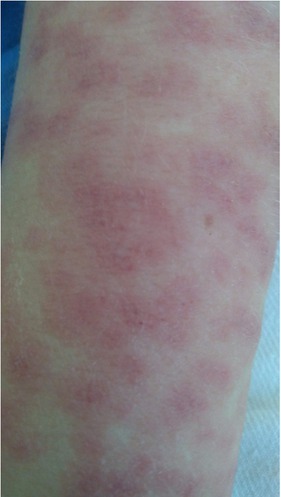
Target shield symptom.

Initially, the patient was treated with cefuroxime and antipyretic drugs. The rash had a tendency to periodically merge, showing the target shield symptom (Figure 1). DRESS Syndrome was suspected. No infection with viruses, including hepatitis A, B and C viruses (HAV, HBV and HCV), human immunodeficiency virus (HIV), cytomegalovirus (CMV) or Epstein–Barr virus (EBV), was confirmed. Abdominal ultrasonography revealed slight enlargement of the liver, spleen and kidneys and the presence of fluid in the peritoneum. The abdominal circumference increased over the following days. Cardiac echocardiography identified fluid in the pericardial sac.

**Figure 2:**
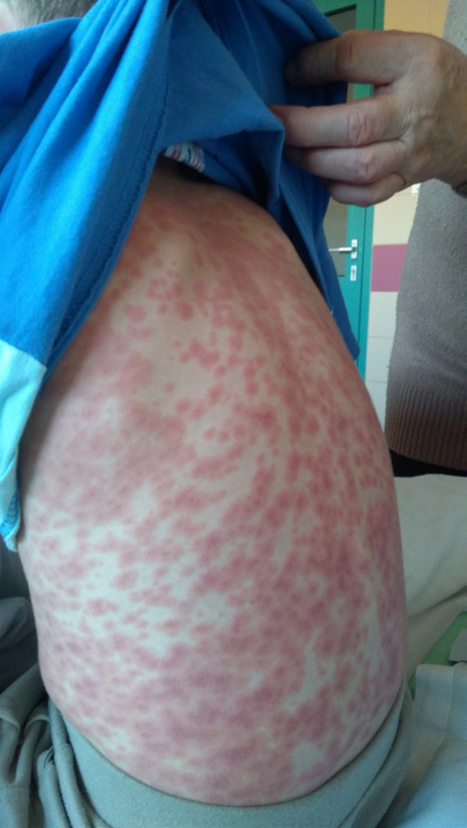
Massive maculopapular lesions covering the skin of the entire body.

Based on the overall clinical picture and the current criteria, DRESS syndrome was diagnosed. On Day 7, methylprednisolone was injected intravenously for 8 days. The patient’s ionic disturbances were equalised, and a single albumin transfusion was administered. Due to the presence of pericardial fluid, spironolactone was given as recommended by the consulting cardiologist. Over the subsequent days, clinical improvement was observed: gradual regression of rash and fever, normalisation of inflammation markers and haemostasis parameters, reduction in the sizes of the liver and spleen and reduction in the amount of fluid in the pericardium and peritoneal sac. The boy was discharged from hospital in a good general condition with the recommendation to continue taking oral prednisone in reducing doses and spironolactone for the next 7 days.

Written informed consent was obtained from the patient and his mother for the publication of this case report.

## Discussion

DRESS is a systemic, severe, drug hypersensitivity syndrome generally observed in adults (3), but it is rarely seen in children (5). Therefore, the presented case is a valuable supplement to the available literature.

DRESS is a drug-induced reaction (3) manifesting as a very wide spectrum of clinical symptoms, including fever >38°C; maculopapular rash, pruritus, erythroderma and exfoliation, occurring mainly on the skin of the face, upper torso and on the extremities, with a tendency to merge within the most intense skin lesions; painful peripheral lymphadenopathy, abdominal pain and involvement of internal organs – liver, kidneys, lungs and heart; and changes in peripheral blood profile (eosinophilia, neutrophilia, neutropaenia, thrombocytopaenia, haemolytic anaemia and presence of atypical lymphocytes in peripheral blood) (3, 5).

Diagnosis is often complicated by the long asymptomatic period between taking the drug and the appearance of symptoms, in addition to the non-specific nature of the symptoms. The most frequently presented symptoms, i.e. fever and rash, are indicative of many childhood diseases, including sepsis. A more specific symptom of DRESS is eosinophilia; however, this is observed in only 30% of patients (6). Due to this variability of the clinical picture, a European criterion for recognising severe cutaneous adverse reactions and drug responses (namely, the Registry of Severe Cutaneous Adverse Reactions or RegiSCAR) has been prepared to facilitate rapid diagnosis (Table 2). (7)

**Table 2:** The RegiSCAR-Group Diagnosis Score for drug rash with eosinophilia and systemic symptoms (DRESS) (12)

Symptom	No	Yes	Unknown
Fever (≥ 38.5°C)	−1	0	−1
Enlarged lymph nodes (>=sites, >=1 cm)	0	1	0
Atypical lymphocytes	0	1	0
Eosinophilia, cells/μL	0		0
700–1,499 or 10%–19.9%		1	
≥ 1,500 or ≥20%		2	
Skin rash	0		0
Extent: >50%	0	1	0
At least two of these: oedema, infiltration, purpura, scaling	−1	1	0
Biopsy suggesting DRESS	−1	0	0
Internal organ involved	0		0
One		1	
Two or more		2	
Resolution in >=15 days	−1	0	−1
At least three biological investigations performed and negative to exclude alternative diagnosis	0	1	0

Final score: >=2 indicates no case; 2–3 indicates possible case; 4–5 indicates probable case; >=5 indicates definite case

The presented patient was awarded seven points according to RegiSCAR, indicating the following: eosinophilia of peripheral blood >=20% (2 points), erythematous rash with accompanying oedema of the lips and ears (2 points), involvement of internal organs (fluid in the pericardial and peritoneal sac) (2 points) and timing (occurrence of the first symptoms 2 weeks after the end of antibiotic therapy), as well as the exclusion of another, alternative diagnosis (1 point).

Although the pathogenesis of the disease is not fully understood, it may be associated with the accumulation of drug metabolites, caused by an innate deficiency of detoxifying enzymes (3). Human leucocyte antigens (HLA) may also play a role, e.g. the HLA-B*1502 phenotype is associated with hypersensitivity to carbamazepine, and the HLA-B*1508 phenotype with hypersensitivity to allopurinol (8). A primary or reactivated viral infection may also be involved (EBV, human herpes viruses 6 and 7 [HHV-6, HHV-7]) (3). Of course, different mechanisms may overlap at the molecular level and complex interactions may be possible.

DRESS may also be associated with the presence of biologically active metabolites of drugs in the blood due to cytochrome dysfunction. Impaired detoxification of these metabolites causes the activation of keratinocytes, macrophages, eosinophils and T-lymphocytes. Upon binding to tissue compatibility antigens on keratinocytes, active drug metabolites stimulate lymphocyte activity, intensify interleukin (IL)-4 and IL-5 production and increase the number of cytotoxic lymphocytes (8, 9).

DRESS syndrome is most often caused by anticonvulsants (carbamazepine, phenytoin, lamotrigine and phenobarbital), anti-tuberculous drugs, allopurinol, sulphasalazine, antibiotics (sulphonamides, vancomycin) and ibuprofen (7).

In our patient, an important factor provoking the development of DRESS syndrome appeared to be vancomycin taken during the initial hospitalisation. Symptoms occurred 2 weeks after the end of vancomycin therapy, which is consistent with the diagnostic criteria of DRESS.

Differentiation of DRESS includes infection with (EBV), (CMV), hepatitis A, B and C viruses (HAV, HBV, HCV); tuberculosis, toxoplasmosis, borreliosis, lambliasis, parvovirus B19 infection, Kawasaki disease, autoimmune diseases, lymphoproliferative diseases, vasculitis, collagenosis, adult Still disease, as well as neoplastic diseases (3). This differential diagnosis was also carried out in our patient.

Treatment consists of discontinuation of the drug that causes the hypersensitivity reaction and implementation of systemic steroid therapy (10). In severe forms resistant to standard treatment, high-dose steroidotherapy (30 mg/kg/day), systemic steroid pulses, intravenous immunoglobulin therapy or plasmapheresis is used (10). The described case is an example of a smooth course of DRESS: the use of steroid therapy in standard doses resulted in the disappearance of symptoms.

## Conclusion

DRESS syndrome is a severe drug-induced hypersensitivity reaction. Diagnosis is complicated by the non-specificity of the clinical picture and the initial asymptomatic period. DRESS should be considered in the diferential diagnosis of children presenting with fever and rash following anticonvulsant or antibiotic treatment. It is extremely important to diagnose this potentially fatal syndrome quickly and implement appropriate treatment.

## Abbreviations

DRESS: drug rash with eosinophilia and systemic symptoms;
HLA: human leucocyte antigens.
